# Depression screening in HIV-positive Tanzanian adults: comparing the PHQ-2, PHQ-9 and WHO-5 questionnaires

**DOI:** 10.1017/gmh.2018.31

**Published:** 2018-11-19

**Authors:** C. P. Nolan, P. J. M. O'Donnell, B. M. Desderius, M. Mzombwe, M. L. McNairy, R. N. Peck, J. R. Kingery

**Affiliations:** 1 Center for Global Health, Weill Cornell Medical College, New York, NY, USA; 2 Department of Medicine, Catholic University of Health and Allied Sciences, Mwanza, Tanzania; 3 Vanderbilt University School of Medicine, Nashville, TN, USA; 4 Department of Medicine, Division of General Internal Medicine, Weill Cornell Medical College, New York, NY, USA; 5 Department of Medicine, Weill Bugando School of Medicine, Mwanza, Tanzania

**Keywords:** Depression screening, etiology, HIV, PHQ-9, Sub-Saharan Africa, WHO-5

## Abstract

**Background.:**

HIV-positive individuals are at significantly increased risk of depression. In low- and middle-income countries, depression is frequently under-detected, hampered by a lack of data regarding available screening tools. The 5-item World Health Organization Well-Being Index (WHO-5) is widely used to screen for depression, yet its validity in African adults with HIV has yet to be examined.

**Methods.:**

In this cross-sectional study, we enrolled HIV-positive adults presenting to an outpatient HIV clinic in Mwanza, Tanzania. Patients were administered the Patient Health Questionnaires (PHQ)-2/9 and WHO-5 questionnaires. The rate of positive screens was calculated. Fisher's exact test and Pearson's correlation coefficients between PHQ-2/9 and WHO-5 scores were calculated.

**Results.:**

We enrolled 72 HIV-positive adults: rates of positive depression screen were 62.5%, 77.8%, and 47.2% according to PHQ-2, PHQ-9, and WHO-5, respectively. PHQ and WHO results for depression were significantly associated (Fisher's exact test: PHQ-2 *v*. WHO-5, *p* = 0.028; PHQ-9 *v*. WHO-5, *p* = 0.002). The level of correlation between PHQ and WHO results for depression was moderate (Pearson's correlation coefficient: PHQ-2 *v*. WHO-5 −0.3289; PHQ-9 *v*. WHO-5 −0.4463).Per Mantel–Haenszel analysis, screening results were significantly more concordant among patients in the following strata: men, age >40, Sukuma ethnicity, Christian, unmarried, self-employed, at least primary school education completed, and higher than the median income level.

**Conclusions.:**

WHO-5 scores correlated well with those of the PHQ-9, suggesting that the WHO-5 represents a valid screening tool. The concordance of PHQ-9 and WHO-5 results was poorer in marginalized socioeconomic groups. Positive depression screens were exceedingly common among HIV-positive Tanzanian adults according to all three questionnaires.

## Introduction

A large body of evidence suggests that people living with HIV (PLWH) are as much as two–four-fold more likely to meet criteria for major depression as compared with HIV-negative individuals. Screening and treatment of depression in PLWH are important as depression is associated with worse medication adherence, morbidity, and mortality (Nanni *et al*., 2015).

Data regarding mental health in low- and middle-income countries (LMIC) remains scarce. Within sub-Saharan Africa, recent epidemiological data suggest that the prevalence of depression is as high or higher than Western populations (Tomlinson *et al*., 2007). Two studies in Uganda have found the prevalence of depression in the rural population to be between 17% and 24% (Bolton *et al*., 2003; Ovuga *et al*., 2005). Studies of depression prevalence within Tanzania have demonstrated variable prevalence, ranging from as low as 3.1% in a general urban population to as high as 23–28% in PLWH, 46.3% among men who have sex with men, and 57% among women living with HIV (Antelman *et al*., 2007; Jenkins *et al*., 2010; Belenky *et al*., 2014; Ahaneku *et al*., 2016; Seth *et al*., 2016). Because depression is common in LMIC and mental health practitioners are few, depressed adults in these countries experience higher rates of mortality secondary to ischemic heart disease and suicide, as well as higher rates of morbidity, with major depressive disorder estimated to account for nearly 1000 years lived with disability per 100 000 people in East Sub-Saharan Africa (Ferrari *et al*., 2013; Kirmayer *et al*., 2017). The vast majority of research on depression has been conducted in high-income countries and most screening and diagnostic techniques have been developed with these populations in mind (Sweetland *et al*., 2014).

The Patient Health Questionnaires (PHQ-2/PHQ-9) and the 5-item World Health Organization Well-Being Index (WHO-5) are two scoring systems which have been widely used for depression screening. The PHQ-2/PHQ-9 has been validated across a number of studies in Africa. Studies in Ethiopia, Nigeria, Kenya, Cameroon, and Uganda have concluded that the PHQ-9 is a valid screening tool with sensitivity and specificity ranging from 86% to 91.6% and 67% to 81.2%, respectively (Adewuya *et al*., 2006; Monahan *et al*., 2009; Pence *et al*., 2012; Akena *et al*., 2013; Gelaye *et al*., 2013; Woldetensay *et al*., 2018). One systematic review of 218 studies utilizing the WHO-5 across the world revealed an average sensitivity and specificity of 0.86 and 0.81, respectively (Topp *et al*., 2015). Although the WHO-5 has been translated into 30 languages and validated in diverse countries such as the USA, Iceland, Japan, China, Norway, and Denmark, it has not yet been validated in Africa (Inagaki *et al*., 2013; Guðmundsdóttir *et al*., 2014; Topp *et al*., 2015; Kong *et al*., 2016; Sirpal *et al*., 2016). Outside of Africa, studies comparing the WHO-5 and PHQ-9 have generally supported the validity of both questionnaires, although their concordance among diverse patient populations is variable (Henkel *et al*., 2004). To the best of our knowledge, no study has yet been published utilizing the WHO-5 questionnaire to screen for depression in PLWH in East Africa or examining the correlation between PHQ-9 and WHO-5 scores in this population.

Therefore, in this cross-sectional study we aimed: (1) to assess correlation between PHQ-9 and WHO-5 scores, (2) to assess concordance between PHQ-9 and WHO-5 scores in different subgroups of PLHIV, and (3) to measure the rate of positive depression screens among PLWH in Tanzania as determined by the PHQ-9, PHQ-2 and WHO-5 screening tools, (4) we hypothesized that PHQ-9 and WHO-5 scores will show strong association and correlation, that the questionnaires will demonstrate higher rates of concordance in non-marginalized subgroups, and that the rate of positive depression screens would be approximately 30%.

## Materials & methods

### Study design

This was a cross-sectional study of 72 PLWH attending an outpatient HIV clinic in Mwanza, Tanzania.

### Study location

This study was conducted in February 2015 in the outpatient HIV clinic at the Bugando Medical Centre (BMC) in Mwanza, Tanzania. BMC is the primary referral hospital for a population of approximately 13 million people in the Lake Victoria Zone in northwest Tanzania. The prevalence of HIV in the catchment area for BMC is approximately 6%, similar to the national average of 5.1%. At the time of the study, BMC was providing outpatient care to 14 432 PLWH, 9064 of whom were on highly-active antiretroviral therapy (HAART).

### Study population

We screened consecutive adults attending the BMC HIV clinic for entry criteria. Inclusion criteria included age >18 years old and HAART naïve. HAART naïve was chosen as an inclusion criterion in order to adhere to Tanzanian practice standards, where patients are typically only screened at their first clinic visit prior to initiation of HAART. Participants with previously diagnosed depression and those who did not speak the national language (Kiswahili) were excluded. A total of 72 clinic patients met the inclusion criteria during the month of February in 2015. None of these were excluded and all 72 agreed to be enrolled in the study.

### Study procedures

At the time of enrollment, each patient completed an extensive socioeconomic questionnaire, followed by the PHQ-9 and WHO-5 questionnaires. Questionnaires were administered by Tanzanian research assistants fluent in Kiswahili. HIV status and medication history were confirmed via clinic medical records. Patients who screened positive for depression on either questionnaire were immediately referred to their HIV clinic physician and the staff psychiatrist for further management according to local medical guidelines.

### Screening tools

The PHQ-2 and PHQ-9 are common depression screening questionnaires that have already been translated and validated in Kiswahili, the national language in Tanzania (Omoro *et al*., 2006). Participants are asked to respond to nine negatively-phrased questions regarding how frequently they have experienced key symptoms and signs of depression over the preceding 2 weeks (ex. ‘Over the last 2 weeks, how often have you had little interest or pleasure in doing things?’). Possible responses and corresponding scores are as follows: not at all (0), several days (1), more than half the days (2), nearly every day (3). The PHQ-2 consists of the first two questions of the PHQ-9, for which a total score of 3 or over is considered a positive depression screening result, warranting more in-depth evaluation with the complete PHQ-9. PHQ-9 scores can be interpreted in a binary (non-depressed *v*. depressed) or multi-level categorical fashion with the following categories and corresponding cut-off scores: non-depressed (0–4), mild (5–9), moderate (10–14), moderately severe (15–19), and severe (over 20). Thus for binary results, any score greater than 4 indicates depression. The WHO-5, is a widely used well-being self-assessment questionnaire and screening tool for depression. We translated the WHO-5 questions into Kiswahili, back translated and then piloted these questions in Kiswahili before the start of the study. Unlike the PHQ-9, respondents to the WHO-5 are asked to agree or disagree with only positively phrased questions (i.e. ‘I have felt cheerful and in good spirits’). Each response and the corresponding score is as follows: at no time (0), some of the time (1), less than half of the time (2), more than half of the time (3), most of the time (4), all of the time (5). Raw scores are typically multiplied by 4, with a total score less than 50 (raw score of 12.5) indicating depression.

### Statistical analysis

All statistical analysis was carried out in Stata. The primary outcome of the study was a correlation between PHQ-9 and WHO-5 screening tool scores as determined by Person's correlation coefficient. Given a two-tailed alpha of 0.05 and medium expected correlation coefficient (r) of 0.30, the sample size was determined to be 75. To determine the significance and degree of correlation between the PHQ-9 and WHO-5 screening tests, the Pearson's correlation coefficient was calculated comparing continuous scores on each scale. Binary screening results of the PHQ-2 and PHQ-9, each *v.* WHO-5 were compared by Fisher's exact test. The five screening levels of the PHQ-9 were compared with the two levels of the WHO-5 via Fisher's exact test. Scores were then converted to a categorical screen result according to aforementioned cut-offs. The Mantel-Haenszel chi-squared test was employed to determine whether concordance between PHQ-9 and WHO-5 differed significantly if patients were stratified according to sex, age, marital status, income level, employment status, religion, or ethnicity. For the purpose of maximizing power, multi-category groups were simplified into binary categories as depicted in Table 1.

**Table 1.** Baseline characteristics of the 72 Tanzanian adult study participants

Variable	
Sex	
Female	47 (65%)
Male	25 (35%)
Age (yrs)	
Median (IQR)	39 (17)
Range	20–62
20–39 years	37 (51%)
40 years and above	35 (49%)
Religion	
Christian	45 (63%)
Other	27 (37%)
Muslim	17 (23%)
Other	10 (14%)
Ethnicity	
Sukuma	25 (35%)
Other^a^	47 (65%)
Marital status	
Married	29 (40%)
Unmarried	43 (60%)
Divorced or widowed	39 (54%)
Never married	4 (6%)
Education level	
Incomplete primary school	21 (29%)
No schooling	12 (17%)
Complete primary school	51 (71%)
Primary school only	41 (57%)
Secondary school	9 (13%)
University	1 (1%)
Employment status	
Self-employed	37 (51%)
Other	35 (49%)
Formal employment	12 (17%)
Unemployed	23 (32%)
Yearly income (TZS)	
Median (IQR)	48500 (82000)
Range	500–800000
0–24999	23 (32%)
25000–49999	13 (18%)
50000–99999	15 (21%)
100000 and above	21 (29%)
CD4^+^ T cell count	
Median (IQR)	668 (252)
Range	46–1500
<200	3 (4%)
200–500	20 (29%)
500–1500	47 (67%)

a Includes Jita (4%), Zinza (3%), Kara (1%), Haya (8%), Luo (7%), Kuria/Shashi (8%), Chaga (1%), other (32%).

### Ethics

The study was approved by the Institutional Review Boards of both Weill Cornell Medicine and BMC. All study participants were informed by a nurse, doctor, or trained staff member fluent in Kiswahili and provided written informed consent prior to participation.

## Results

### Enrollment characteristics

A total of 72 people were enrolled in the study. As detailed in Table 1, 65% of patients were female. The median age of study participants was 39 years (interquartile range: 30–47). In addition, 35% were from the Sukuma tribe (the majority tribe in Mwanza); 63% identified as Christian; 71% of patients had completed primary school; and 51% of patients described themselves as self-employed. The median monthly income for the cohort was 48 500 Tanzanian Shillings (~$20 per month).

### Screening results

As depicted in Table 2, 62.5% of PLWH screened positive for depression based on the PHQ-2, 77.8% based on the PHQ-9, and 47.2% based on the WHO-5. The most common screening result for the PHQ-9 was moderate depression (27.8%).

**Table 2.** Prevalence of depression

Screening questionnaire	Screening result	N (%)
PHQ-2	Non-depressed	27 (37.5)
	Depressed	45 (62.5)
PHQ-9	Non-depressed	16 (22.2)
	Depressed	56 (77.8)
	Mild	18 (25)
	Moderate	20 (27.8)
	Moderately severe	14 (19.4)
	Severe	4 (5.6)
WHO-5	Non-depressed	38 (52.8)
	Depressed	34 (47.2)

### Association between PHQ and WHO-5 questionnaires

Fisher's exact test comparing depression diagnosed by PHQ-2 and WHO-5 revealed a statistically significant association between the two tests, *p* = 0.03 (Table 3). Depression diagnosed by PHQ-9 and WHO-5 was also significantly associated, *p* = 0.002 (Table 4). Fisher's exact test comparing the five levels of depression outcomes by the PHQ-9 and the two levels of the WHO-5 also revealed a statistically significant association, *p* = 0.008 (Table 5).

**Table 3.** Fisher's exact test comparing PHQ-2 and WHO-5 results

	WHO-5 categorical screening result		
PHQ-2 categorical screening result	Depressed (%)	Non-depressed (%)	Total
Depressed	26 (57.78)	19 (42.22)	45
Non-depressed	8 (29.63)	19 (70.37)	27
Total	34 (47.22)	38 (52.78)	72

Fisher's exact test = 0.028.

Pearson's correlation = 0.2730 (*p* < 0.0203).

**Table 4:** Fisher's exact test comparing PHQ-9 (binary) and WHO-5 results

	WHO-5 categorical screening result		
PHQ-9 categorical screening result	Depressed (%)	Non-depressed (%)	Total
Depressed	32 (94.12)	2 (5.88)	34
Non-depressed	24 (63.16)	14 (36.84)	38
Total	56 (77.78)	16 (22.22)	72

Fisher's exact test = 0.002.

Pearson's correlation = 0.3718 (*p* < 0.0013).

**Table 5.** Fisher's exact test comparing PHQ-9 (multi-level) and WHO-5 results

	WHO-5 categorical screening result		
PHQ-9 categorical screening result	Depressed (%)	Non-depressed (%)	Total
Non-depressed	2 (12.50)	14 (87.50)	16
Mild depression	8 (44.44)	10 (55.56)	18
Moderate depression	14 (70)	6 (30)	20
Moderately severe depression	8 (57.14)	6 (42.86)	14
Severe depression	2 (50)	2 (50)	4
Total	34 (47.22)	38 (52.78)	72

Fisher's exact test = 0.008.

Pearson's correlation = 0.3104 (*p* < 0.0080).

### Correlation between PHQ and WHO-5 questionnaires

The Pearson's correlation coefficient between the PHQ-2 and PHQ-9 numerical scores was 0.6667 (*p* < 0.0001), indicating a strong correlation. The Pearson's correlation coefficient between PHQ-2 and WHO-5 numerical scores was −0.3289 (*p* < 0.0048), indicating a moderate correlation. The Pearson's correlation coefficient between PHQ-9 and WHO-5 numerical scores was −0.4463 (*p* < 0.0001), indicating a moderate correlation. A negative correlation was expected, given that the WHO-5 measures well-being and PHQ-9 measures depression symptoms. Both PHQ and WHO-5 data exhibited normal distribution.

### Concordance of PHQ-9 and WHO-5 results among sociodemographic strata:

According to Mantel-Haenszel chi-square analyses, men, as compared with women, were significantly more likely to have concordant screening results (χ^2^ = 9.59, *p* = 0.002). Patients over 40 years of age, as compared with those younger, were significantly more likely to have concordant screening results (χ^2^ = 8.77, *p* = 0.003). Sukuma patients, as compared with all other ethnic groups, were significantly more likely to have concordant screening results (χ^2^ = 9.82, *p* = 0.002). Unmarried patients (divorced, widowed, or never married) were significantly more likely, as compared with married patients, to have concordant screening results (χ^2^ = 9.73, *p* = 0.002). Christian patients, as compared with patients with any other religious affiliation, were significantly more likely to have concordant screening results (χ^2^ = 9.58, *p* = 0.002). Patients who had at least completed primary school, as compared with those with either no schooling or incomplete primary school, were significantly more likely to show concordant screening results (χ^2^ = 10.23, *p* = 0.0014). Self-employed patients, as compared with those with no employment or other forms of employment, were significantly more likely to have concordant screening results (χ^2^ = 10, *p* = 0.0016). Finally, patients with income of 50 000 TZS per year or greater were significantly more likely to show concordant screening results as compared with patients with lower income (χ^2^ = 9.56, *p* = 0.002).

## Discussion

The WHO-5 likely represents a useful depression screening tool for PLWH in Tanzania. These data demonstrate a significant association and moderate correlation between WHO-5 and PHQ-9 scores. While the PHQ-9 has been previously validated among PLWH in east Africa, we report the first correlation in this region with the WHO-5 questionnaire. This may have important implications for depression screening in settings with limited mental health staff, limited resources and a lack of available screening tools. While this correlation is important, more robust validation of multiple depression screening tools is needed in order to strengthen the screening toolbox of Tanzanian primary care practitioners.

Concordance between WHO-5 and PHQ-9 scores revealed that screening results varied across demographic strata. This suggests that depression screening questionnaires correlate heterogeneously among Tanzanian HIV-infected adults in nuanced ways even within the same cultural setting. This is unsurprising given that prior studies comparing WHO-5 and PHQ-9 questionnaires have demonstrated variability between scores within similar but geographically distinct populations (Henkel *et al*., 2003; Löwe *et al*., 2004). Additionally, such variability exists in a disease-specific manner, suggesting that more nuanced factors likely affect the performance of screening questionnaires (Lloyd *et al*., 2012; Wu, 2014; Englbrecht *et al*., 2017). Our results suggest that the WHO-5 correlates best with the PHQ-9 in Tanzania among men, those older than 40, members of the Sukuma tribe, Christians, and those with higher education and income levels. This distinction is especially important as groups showing poor concordance are also the same groups which are typically at highest risk of depression – women, the uneducated, the impoverished, and members of minority religious and ethnic groups. Identification, or development, of valid and reliable screening tools for these especially vulnerable members of the population, are needed if the burden of depression in Tanzania and other African countries is to be truly ameliorated.

Rates of positive depression screens were exceedingly common among Tanzanian PLWH by all three measures utilized (PHQ-2, PHQ-9, and WHO-5). The highest, 77.8% according to the PHQ-9, is significantly higher than previous estimates of 17–24% in a general East African population, in agreement with the two–four-fold higher risk among PLWH that has been reported (Nanni *et al*., 2015; Bolton *et al*., 2003; Ovuga *et al*., 2005). This is also higher than prior estimates among PLWH in other parts of sub-Saharan Africa. Studies of PLWH in Uganda, Zimbabwe, Cameroon, South Africa, and Kenya report highly variable rates of depression among PLWH, ranging widely from 3% to 68.5% (Monahan *et al*., 2009; Pence *et al*., 2012; Akena *et al*., 2013; Cholera *et al*., 2014; Chibanda *et al*., 2016*a*, *b*). The high rate of positive depression screens in our cohort, consistent across multiple measures, suggests that the burden of depression among PWLH in East Africa may be more significant than previously thought. This said, validation with a gold standard diagnostic tool is needed to determine true prevalence.

The rate of positive depression screens as measured by the PHQ-2 in this study was 15.3% less than that measured by the PHQ-9. Additionally, the correlation between PHQ-2 and WHO-5 was, while significant, overall weaker than that between PHQ-9 and WHO-5. This suggests that the first two questions of the PHQ-9 may not adequately capture the symptoms experienced by depressed Tanzanians as well as the remainder of the questionnaire, a finding previously reported in other areas (Hanlon *et al*., 2015). The Operational Manual for Comprehensive Differentiated Delivery of HIV and AIDS Services distributed by the National AIDS Control Programme of Tanzania currently recommends initial screening of PLWH with the PHQ-2 (Ministry of Health, 2017). These results, however, suggest that a significant portion of Tanzanian PLWH suffering from depression would remain undetected unless the full PHQ-9 questionnaire, or an equivalent such as the WHO-5, is administered.

There are limitations to the current study. This study was designed to correlate two depression screening tools; therefore, the validity of each screening tool as compared with gold standard diagnostic tools and subsequent true prevalence of depression cannot be determined. Additionally, while we report the first correlation of the WHO-5 as compared with any other depression screening tool in east Africa, a possibility of intraregional variation remains. Our population of HIV-infected adults at a city-based, tertiary referral hospital has not been correlated to more rural populations.

In conclusion, we conducted a cross-sectional study of 72 outpatient, Tanzanian PLWH utilizing the PHQ-2, PHQ-9, and WHO-5 screening questionnaires to assess the correlation of scores among different questionnaires, concordance across sociodemographic strata, and rates of positive depression screens. Our results suggest that the WHO-5 represents a useful tool in Tanzania, and potentially East Africa. This tool performed most homogenously among older, male, Sukuma patients with higher education and income. Rates of positive depression screens were high, suggesting that depression is an under-detected and under-treated comorbidity among PLWH in Tanzania. Importantly, none of the patients with positive screens in this study had been previously diagnosed or screened for depression. Studies indicate poor clinical outcomes among PLWH with untreated depression. This is worsened by the current lack of psychiatrists in Tanzania, thereby shifting the burden of screening and diagnosis to primary care staff. Therefore, the addition of another screening questionnaire such as the WHO-5 to their toolbox may be invaluable. Integration of regular depression screening into HIV clinic standard care and training of primary care providers in identification and treatment of depression may represent a viable avenue toward improving the long-term health of this population.
